# EGFR activation differentially affects the inflammatory profiles of female human aortic and coronary artery endothelial cells

**DOI:** 10.1038/s41598-023-50148-7

**Published:** 2023-12-20

**Authors:** Virginie Dubourg, Gerald Schwerdt, Barbara Schreier, Michael Kopf, Sigrid Mildenberger, Ralf A. Benndorf, Michael Gekle

**Affiliations:** 1 Julius-Bernstein-Institute of Physiology, Martin Luther University Halle-Wittenberg, Magdeburger Str. 6, 06112 Halle, Germany; 2 Department of Clinical Pharmacy and Pharmacotherapy, Institute of Pharmacy, Martin Luther University Halle-Wittenberg, Halle, Germany

**Keywords:** Vascular diseases, Cell signalling, High-throughput screening

## Abstract

Endothelial cells (EC) are key players in vascular function, homeostasis and inflammation. EC show substantial heterogeneity due to inter-individual variability (e.g. sex-differences) and intra-individual differences as they originate from different organs or vessels. This variability may lead to different responsiveness to external stimuli. Here we compared the responsiveness of female human primary EC from the aorta (HAoEC) and coronary arteries (HCAEC) to Epidermal Growth Factor Receptor (EGFR) activation. EGFR is an important signal integration hub for vascular active substances with physiological and pathophysiological relevance. Our transcriptomic analysis suggested that EGFR activation differentially affects the inflammatory profiles of HAoEC and HCAEC, particularly by inducing a HCAEC-driven leukocyte attraction but a downregulation of adhesion molecule and chemoattractant expression in HAoEC. Experimental assessments of selected inflammation markers were performed to validate these predictions and the results confirmed a dual role of EGFR in these cells: its activation initiated an anti-inflammatory response in HAoEC but a pro-inflammatory one in HCAEC. Our study highlights that, although they are both arterial EC, female HAoEC and HCAEC are distinguishable with regard to the role of EGFR and its involvement in inflammation regulation, what may be relevant for vascular maintenance but also the pathogenesis of endothelial dysfunction.

## Introduction

Endothelial cells (EC) are the innermost layer of blood vessels, playing thus a critical role in supplying oxygen and nutrients to the organs, in waste removal from the latter and in immune cell trafficking. They are key players in vascular function, homeostasis and inflammation. EC reportedly display organ-specific functions and phenotypes to fulfil the different physiological needs of the said organs. For instance, lung EC are specialized for efficient gas exchange at the blood-air barrier, while heart EC display preferential fatty acid uptake and ensure their supply to cardiomyocytes, which rely on fatty acids for cardiac contraction^1^. Various studies additionally report variations in gene expression in between EC from different organs^2–7^. However, most of these studies compared EC from whole organs, which arguably correspond to a mix of EC from different vascular beds and mostly to microvascular EC (from capillaries)^3^. The extrapolation of results from microvascular EC to macrovascular EC (e.g., from arteries) is questionable due to distinct gene expression profiles in EC from large vessels and microvascular EC^4^. Nevertheless, variations of gene expression in between different types of macrovascular EC have hardly been reported yet^8–12^. In either case, previous studies rarely took into consideration the sex of the donors, especially in studies based on human samples, most probably due to the difficulty to obtain such samples from patients without biasing pathologies (e.g. atherosclerosis, hypertension). However, intrinsic sex differences in endothelial cells from males and females have been reported, at the functional and transcriptional levels^13–15^, what may have thus influenced the drawn conclusions.

Here we wanted to compare the responsiveness of macrovascular female human primary EC from the aorta (HAoEC) and coronary arteries (HCAEC) to a cardiovascular-relevant mediator. HAoEC and HCAEC are from vessels deriving from distinct stages of the embryonic angiogenesis^16^ and from structurally different vessels. Indeed, although they are all vessels subject to high blood pressure, the aorta is a large diameter conductance-type vessel and an elastic artery, while coronary arteries are resistance-type vessels and muscular arteries. These structural differences result in a heterogeneous arterial compliance throughout the vascular tree, which is further strengthened by differential endothelial and VSMC functions in both artery types^17^. Additionally, conductive and resistant vessels differ in pathological situations, such as hypertension and in the related patterns and mechanisms of vascular remodeling^18^. Taken together, this suggested putative differences between HAoEC and HCAEC, including with regards to responsiveness to external stimuli.

For our investigation, we opted for the stimulation of the Epidermal Growth Factor Receptor (EGFR), an important signalling-hub associated with vascular physiology and pathophysiology. Indeed, ligand-dependent EGFR activation controls various signalling modules, thereby affecting transcriptional regulation and finally e.g. cell proliferation, survival, differentiation, migration and matrix homeostasis^19^. But EGFR can also be transactivated by receptors for vasoactive substances (e.g. Angiotensin II), thereby contributing to vascular tone, dysfunction and remodelling as a transducer for non-EGFR ligands^20^. The relevance of EGFR in vascular smooth muscle cells (VSMC), the major cell type in arterial walls, and in cardiovascular health and disease has been shown^21–24^. While the importance of endothelial EGFR (EC-EGFR) is not yet as well understood, there is evidence for a modulatory role of EC-EGFR in normal tissue besides its enhanced expression in tumour endothelium^25^, in the regulation of basal vascular function^26^ or during the development of endothelial dysfunction^21^. The comparison of the responsiveness of HAoEC and HCAEC to EGFR stimulation with EGF is therefore relevant for cardiovascular pathophysiology.

Therefore, we stimulated HAoEC and HCAEC with 10 ng/mL EGF, a concentration comprised in the in vivo pathophysiological range^27^, and evaluated the impact on gene expression and cellular phenotypes. To do so, we first performed a transcriptomic analysis using newly generated RNA-sequencing datasets, with samples from one donor only for each cell type, but with high statistical powers (each group comprised 8 independent biological replicates). Based on these data, we generated the hypothesis that EGF partly triggered a cell-type specific effect, not only quantitatively (number of regulated genes) but also qualitatively (putatively affected cellular functions). Indeed, further analysis suggested that the EGF-induced changes in gene expression associated with a cell type-specific regulation of inflammation. Since at that point we could not determine if these differences were attributable to the cell types themselves or to the inter-individual variability between the two donors, we measured selected inflammation markers (e.g. cytokine and adhesion molecule expression, leukocyte adhesion) in HAoEC and HCAEC from multiple female donors to experimentally confirm some of the hereinabove bioinformatics predictions. The results showed that EGF led to the regulation of these markers in a cell type-dependent but donor-independent manner, and that EGF may play a protective role in HAoEC while playing a detrimental one in HCAEC. Finally, we compared the two sets of RNA-sequencing data and identified processes that could be differentially regulated in HAoEC and HCAEC under basal conditions.

## Results

### EGFR activation leads to various gene expression regulation patterns in EC

**Figure 1:**
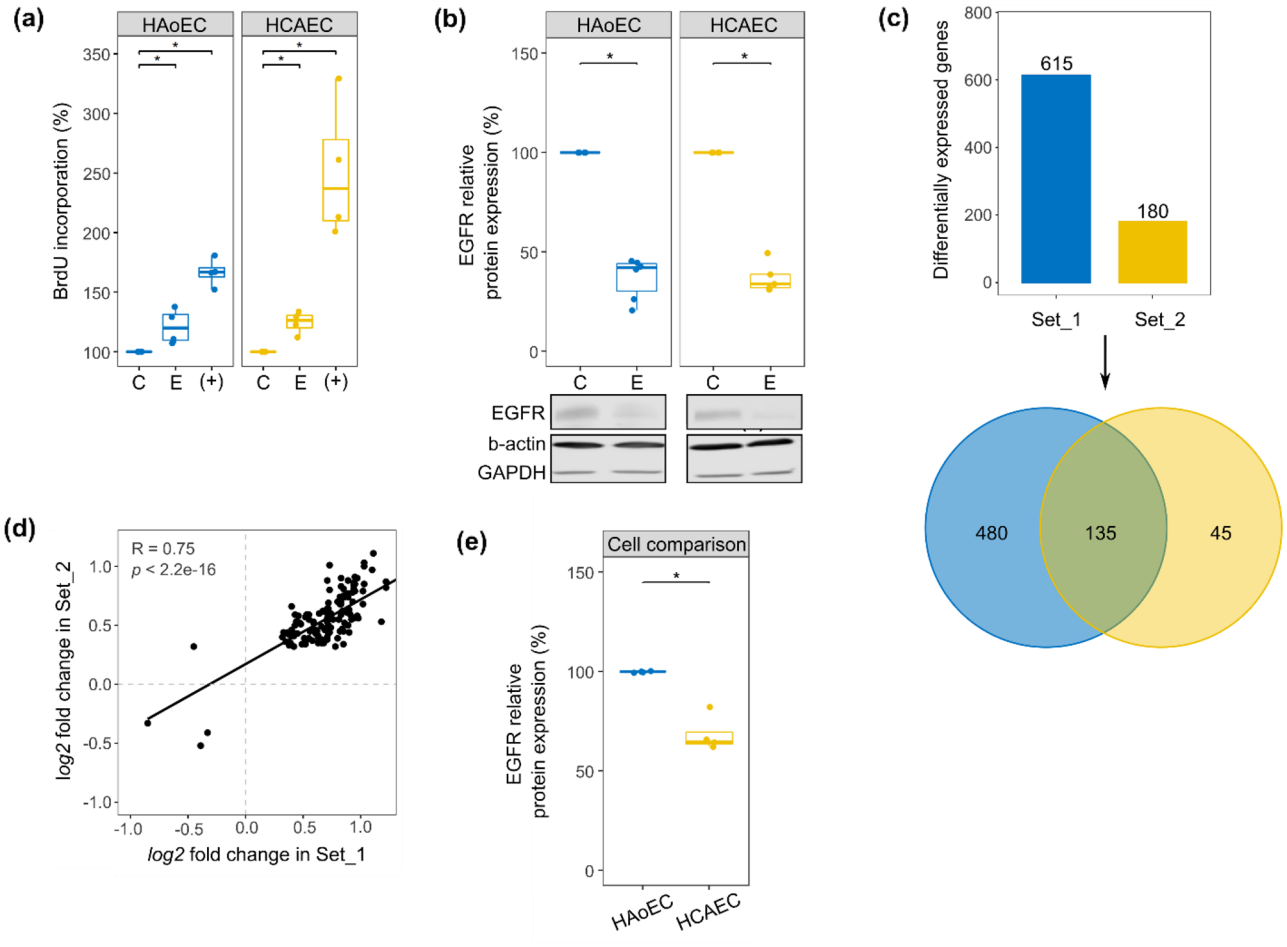
Response to EGFR activation in HAoEC and HCAEC. (**a**) Responsiveness to EGF (E) was estimated using BrdU incorporation, a marker for DNA synthesis and therefore cell proliferation. The control condition (C—media with minimal supplementation—see “Methods”) was used as reference for each independent replicate (number of independent replicates N = 4). The fully supplemented media (used for usual cultivation of EC) served as positive control (+). (**b**) EGFR relative protein expression after 48 h incubation with EGF (N = 5). Representative cut membranes are shown here but whole Western Blot membranes are presented in Supplementary Fig. 3. (**c**) Number of genes regulated in Set_1 (comprising samples from HAoEC from one donor) and Set_2 (comprising samples from HCAEC from one donor) following stimulation with EGF and the result of the comparison of two lists of regulated genes (Venn diagram). (**d**) Scatter plot depicting the log2 fold changes calculated in Set_1 and Set_2 for the genes regulated in both sets by EGF (genes comprised in the overlap of the Venn diagram, Fig. 1c). The correlation score (R) was calculated using Pearson’s method. (**e**) EGFR relative protein expression when comparing the two cell types. HAoEC were used as reference and unstimulated samples from HAoEC and HCAEC were randomly paired for comparison (N = 4). The results of the BrdU incorporation assay and the Western Blots were obtained using cells/samples from multiple female donors for each cell type. * Wilcoxon test, *p* < 0.05.

We aimed to investigate if aortic and coronary artery EC have the same level of responsiveness to EGFR activation by EGF. We first verified if such cells were responsive to EGF at all. To do so, we stimulated HAoEC and HCAEC from multiple female donors and measured (1) DNA synthesis as EGF is described as a promotor of cell proliferation^22^ and (2) the total amount of EGFR after incubation with EGF since this receptor is known to be downregulated following its stimulation^28^. EGFR activation triggered an increase in DNA synthesis and a decrease of the total amount of EGFR in both cell types, in a donor-independent manner (Fig. 1a,b).

As HAoEC and HCAEC appeared responsive to EGF, we proceeded in evaluating the effect of long-term EGFR activation on their transcriptome (measured after 48 h stimulation with EGF). RNA-sequencing was performed for 2 sets of samples with high statistical power (number of independent biological replicates N = 8). Set_1 comprised samples from HAoEC from a single donor and Set_2 comprised samples from HCAEC from a different single donor. Differential expression analysis showed that 615 and 180 genes were regulated by EGF in Set_1 and Set_2, respectively (Fig. 1c and Supplementary Table S1). 135 genes were regulated in both sets, although with different regulation amplitude (Figs. 1c,d). Assuming this difference in number of regulated genes was not only due to the variability between the two donors but actually attributable to inherent differences between the cell types, we checked the basal expression level of EGFR in HAoEC and HCAEC by Western Blot, using samples from multiple donors for each cell type (Fig. 1e). HCAEC appeared to express EGFR at a lower level than HAoEC, what could partly explain the difference observed when comparing Set_1 and Set_2.

**Figure 2:**
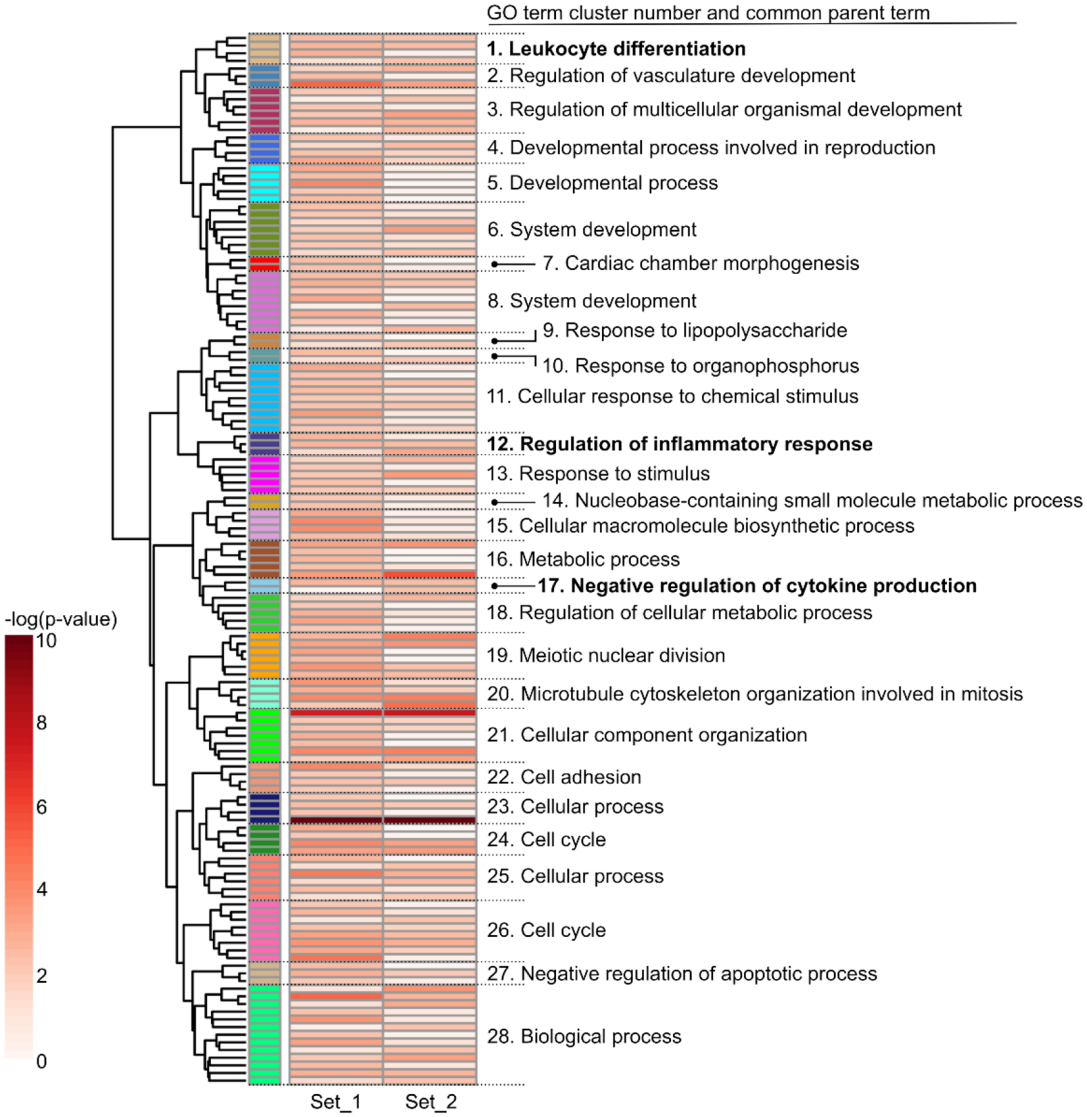
Stimulation of vascular EC with EGF triggers the regulation of genes involved in different processes. Heatmap (generated using ViSEAGO R package—see “Methods”) showing GO terms that were significantly enriched for at least one list of regulated genes used as input for the analysis. Each row corresponds to a GO term and the colour gradient to their respective − log (*p*-value) (the detailed list of GO terms and the corresponding *p*-values are available in Supplementary Table S2). The GO term clusters are indicated by different colours and numbers. The common parent term of the GO terms comprised in a given cluster is indicated. Those associated with inflammation regulation are highlighted in bold.

Gene ontology (GO) term enrichment analysis served to identify biological processes that may be affect by EGFR activation. The lists of differentially expressed genes in Set_1 and Set_2 were used as input. The GO terms significantly enriched with genes from at least one of these two lists were considered for clustering (Fig. 2 and Supplementary Table S2). Based on the tree-like organization of gene ontology, the common parent term of the included GO terms was identified for each cluster. Unsurprisingly, this annotation showed that some clusters of enriched GO terms were associated with cell cycle and proliferation (clusters 19, 20, 24 and 26), with cell survival (cluster 27) or yet with vasculature development (cluster 2), processes associated with angiogenesis and in which EGFR is already known to play a role^22^. However, some clusters of enriched GO terms were also associated with inflammation regulation (cluster 1, 12 and 17), suggesting an effect of EGF on this process as well.

### EGF differentially regulates inflammation-related genes in distinct EC

**Figure 3:**
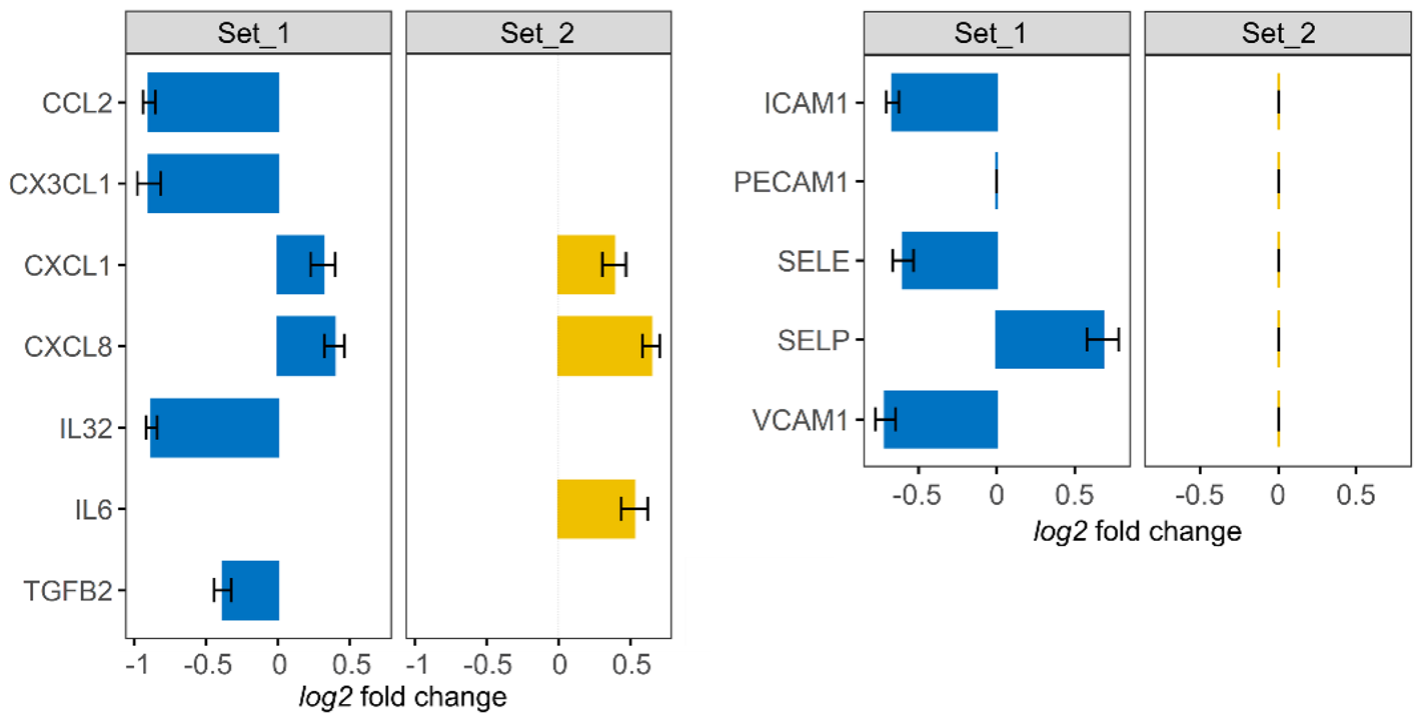
EGF leads to different cytokine/chemokine and adhesion molecule expression profiles in different endothelial cells. Log2 fold changes (calculated by DESeq2, error bars show the standard errors) for significantly differentially expressed cytokines/chemokines (left) and selected adhesion molecules (right) following EGF-incubation (Supplementary Table S1).

Since the bioinformatics analysis results suggested an effect of EGFR activation on inflammation regulation, we checked if fundamental inflammation mediators were regulated in Set_1 and Set_2 following EGF stimulation. The EGF-regulated genes (Supplementary Table S1) were screened for cytokines and chemokines (using IL*, CX*, CSF*, TGFB*, IFN*, MST*, CCL* as search keys). 6 and 3 cytokine/chemokine coding-genes were found significantly regulated in Set_1 and Set_2, respectively (Fig. 3). All coded for pro-inflammatory cytokines/chemokines. Out of these, only *CXCL8* (IL-8) and *CXCL1* were regulated in both sets, while the other ones appear to be regulated in a set-specific manner. The genes regulated specifically in Set_1 were all downregulated, while those regulated specifically in Set_2 were upregulated by EGF. If assuming that each set actually reflected what happened in its corresponding cell type, these results suggest that EGF led to a cell type-specific regulation of cytokine/chemokine expression.

Additionally, we checked for expression changes of genes coding for adhesion molecules that are critical for endothelium integrity and immune cell adhesion^29^, such as *SELE* (Selectin E), *SELP* (Selectin P), *VCAM1* (vascular cell adhesion molecule 1), *ICAM1* (intercellular adhesion molecule 1) and *PECAM1* (platelet/endothelial cell adhesion molecule 1) (Fig. 3). None were significantly regulated by EGF in Set_2. On the other hand, *SELP* was upregulated and *SELE*, *VCAM1* and *ICAM1* were downregulated by EGF in Set_1. Here again, if considering that the results obtained for each set are representative of the corresponding cell type, they suggest that EGF led to a cell type-specific regulation of adhesion molecule expression.

Taken together, these results indicated that EGFR activation led to a distinct regulation of genes involved in inflammation-related processes in Set_1 (HAoEC) and Set_2 (HCAEC). Therefore, EGFR activation may trigger an arterial cell type-specific inflammation regulation, more specifically with a putative anti-inflammatory response in HAoEC but a pro-inflammatory one in HCAEC.

### EGFR activation may favour leukocytes accumulation to EC

Further analysis with the IPA software was performed in order to specify which inflammatory-related cellular processes may be affected by the EGF-induced gene expression regulations (in a set-/cell type-specific manner or not). To do so, we first used the IPA “Diseases and Functions” tool for the genes regulated in Set_1 and in Set_2. Similarly to GO term enrichment analysis, this tool performs downstream analysis and helps to generate hypotheses concerning which cellular functions may be affected by gene expression regulation. The output of this first step was used for the IPA “Comparison Analysis” option, which matched functions that were significantly enriched for at least one set of regulated genes. The latter were filtered for inflammation-related functions. The results evoke an influence of EGF on leukocytes attraction and accumulation by EC (Table 1). The “Ingenuity Downstream Effects Analysis in IPA” guidelines recommend to use |Z-score|≥ 2 as significance threshold when working with “unbiased” data sets (approximately equal number of up- and down-regulated genes) (pages.ingenuity.com/rs/ingenuity/images/0812%20downstream_effects_analysis_whitepaper.pdf). However, EGF incubation led mostly to upregulation of gene expression (Supplementary Fig. 2), what calls the use of this strict threshold in question. Scaling down this threshold, the function “Accumulation of leukocytes” appeared putatively activated, preferentially in Set_2 (Z-score = 1.88) rather than in Set_1 (1.08). The function “Cellular infiltration by leukocytes”, while displaying high − *log*(B-H *p*-values), was not predicted as activated nor inhibited in either cell type. The same applies for “Cell movement of leukocytes” and “Leukocyte migration” functions in Set_1.

**Table 1:** EGF may influence the adhesion of leukocytes to EC.

Diseases and bio functions	Set_1		Set_2	
	z-score	− *log* (B-H *p*-value)	z-score	− *log* (B-H *p*-value)
Accumulation of leukocytes	1.08	7.54	1.88	5.13
Cell movement of leukocytes	− 0.23	9.30	N/A	0.00
Cellular infiltration by leukocytes	− 0.17	9.25	0.19	4.36
Leukocyte migration	0.29	10.88	N/A	0.00

Output of the “Comparison analysis” by IPA for “Diseases and functions” filtered for inflammation-related processes. Functions enriched (− log (BH (Benjamini-Hochberg) *p*-value) ≥ 1.3) for at least one cell type are listed. Z-scores were calculated based on annotations in the software internal database. Positive and negative Z-scores correspond to putatively promoted and inhibited functions, respectively. N/A stands for undetermined Z-scores because there was no enrichment for this function for a given dataset (− log (BH *p*-value) = 0).

**Figure 4:**
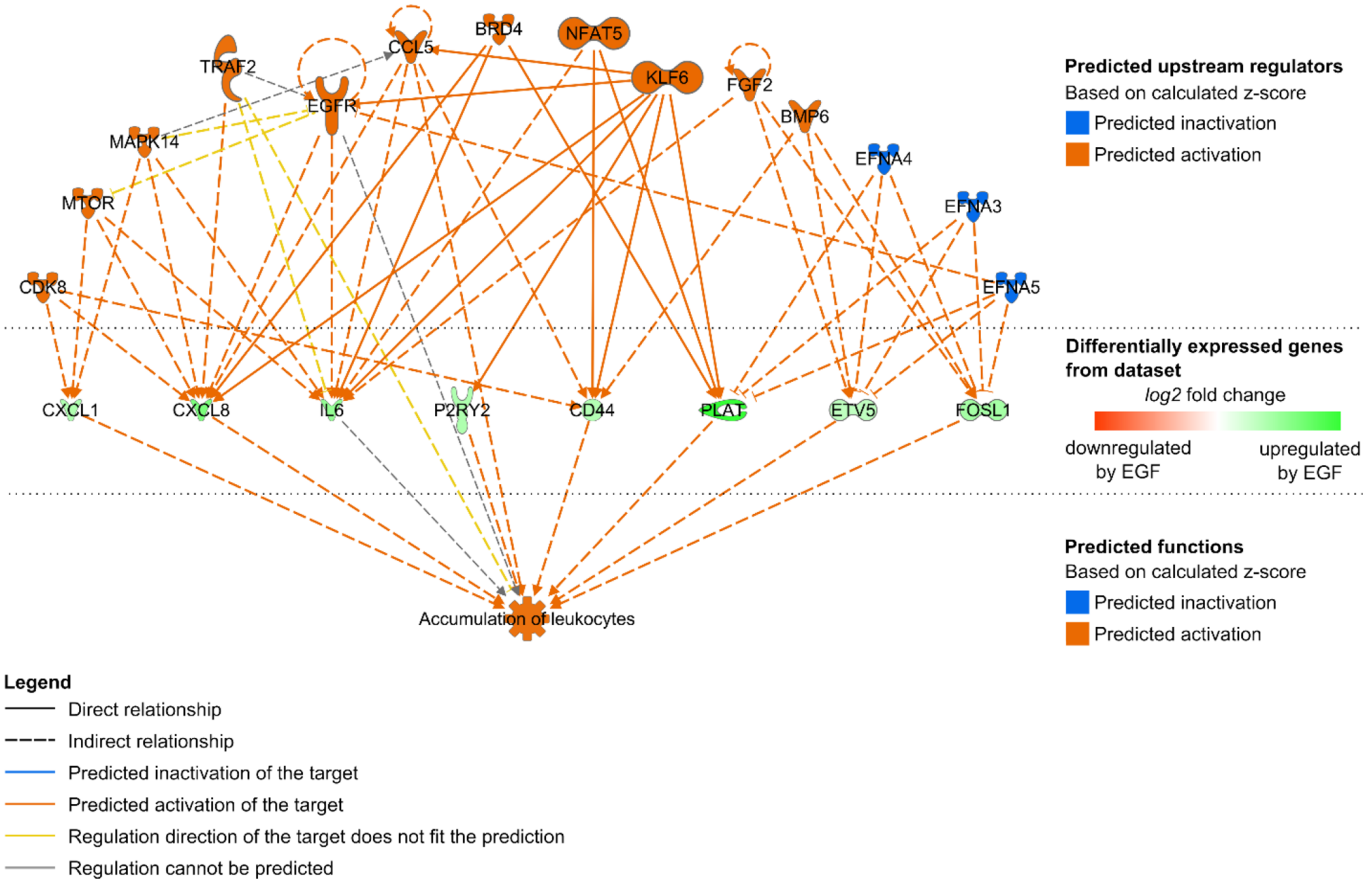
Network showing putative underlying mechanisms leading to leukocyte accumulation following EGF stimulation. IPA “Regulator Effects” consists in connecting predicted upstream regulators, regulated genes (input dataset) and predicted affected downstream functions. The actual network shows the predicted upstream regulators (top) of the EGF-regulated genes in Set_2 (middle) that have been associated with the “Accumulation of leukocytes” function (bottom).

**Figure 5:**
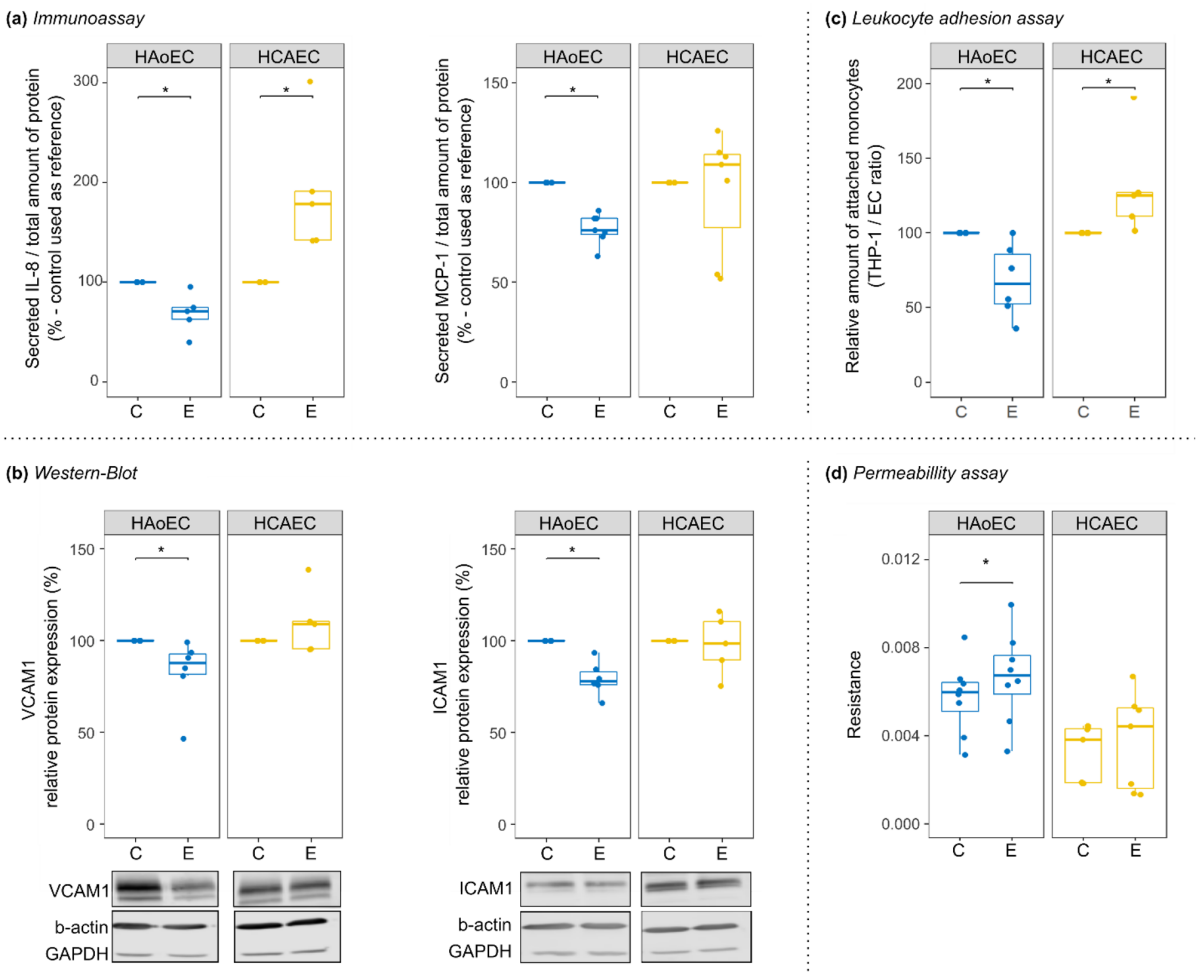
EGFR activation regulated inflammation biomarkers in a cell type-dependent manner. The effect of EGF-incubation (E) was compared to control conditions (C) for several parameters. Samples were prepared from cells from multiple donors for each cell type. (**a**) The concentrations of the pro-inflammatory cytokines IL-8 (number of independent replicates N = 5) and MCP-1 (N = 7) were measured by immunoassay. (**b**) The expression level of the adhesion molecules VCAM-1 and ICAM-1 were measured by Western-Blot (N = 6). (Representative cut membranes are shown here but whole membranes are displayed in Supplementary Fig. 3). Finally, to estimate the consequences of changes in some of the hereinabove inflammation biomarkers, we performed (**c**) a leukocyte adhesion assay (N = 5–6) and (**d**) a permeability assay (N = 7–8). For each experiment performed to evaluate the effect of EGF, the control condition (media with minimal supplementation) was used as reference for each independent replicate. * Wilcoxon test, *p* < 0.05.

A network was generated with the help of the IPA “Regulator Effects” tool to identify the regulated genes and their putative upstream regulators that may be involved in the presumably enhanced accumulation of leukocytes following EGF-stimulation (Fig. 4). Indeed, the idea behind IPA “Regulator Effects” is to connect predicted upstream regulators, the corresponding regulated genes (result of the differential expression analysis used here as input) and downstream functions that may be affected by the latter. To do so, its algorithm merges the results of upstream and downstream analysis (https://resources.qiagenbioinformatics.com/white-papers/Regulator_Effects_in_IPA.pdf). This allows to generate hypotheses to explain how the activation or inhibition of certain upstream regulators may impact biological functions via gene expression regulation. A Consistency Score (CS) is calculated for each generated network that rewards consistent regulator-target-function connections, meaning that networks with higher CS represent more consistent hypotheses. Networks associated with an enhanced “accumulation of leukocytes” were found for Set_1 and Set_2, with CS = 6.05 and 11.67, respectively (Supplementary Table S3). Once again, this result suggests a more likely tendency for leukocyte accumulation by the cells from Set_2 than by those in Set_1. Additionally, it is worth noting that EGFR stands among the predicted activated upstream regulators of the leukocyte accumulation by the cells from Set_2 (Fig. 5), implying that this inflammation-related consequence could then directly be related to the applied stimulus and not just a side effect to it.

### EGF triggers opposite inflammatory responses in aortic and coronary endothelial cells—experimental confirmation

We predicted that EGFR activation led to a differential regulation of inflammation markers in the cells used to generate Set_1 (HAoEC) and Set_2 (HCAEC), with results hinting at anti- and pro-inflammatory regulations, respectively. However, up to that point, we could not distinguish if these differences were due to the donor variability or to an actual differential effect of EGF on the two cell types. Thereby, to go beyond bioinformatics, we experimentally checked for inflammation-related phenotypic traits that were predicted to be influenced by EGF. To do so, we used HAoEC and HCAEC from multiple female donors. Based on the results above, we first focused on factors that may influence leukocyte attraction to the endothelium such as cytokine secretion and adhesion molecule expression.

We used immunoassays to measure the amount of secreted Interleukin 8 (IL-8, encoded by *CXCL8*) and Monocyte Chemoattractant Protein 1 (MCP-1, encoded by *CCL2*) in cell culture media. They are known pro-inflammatory cytokines associated with neutrophil and monocyte recruitment, and key players in the monocyte-endothelium interactions^30^. Our RNA-sequencing data identified these cytokines as regulated by EGF (Fig. 3). The regulation of IL-8 by EGFR activation in both cell types was confirmed (Fig. 5a). However, while IL-8 secretion by HCAEC increased upon EGFR activation, its secretion by HAoEC decreased, showing a discrepancy with the RNA-sequencing results obtained with Set_1 (containing HAoEC). On the other hand, the HAoEC-specific downregulation of MCP-1 was confirmed (Fig. 5a).

Additionally, the protein expression levels of VCAM1 and ICAM1 were assessed by Western Blot, as our RNA-sequencing results suggested a HAoEC-specific downregulation of these adhesion molecules following EGFR activation (Fig. 3). This cell type-specific EGF-induced downregulation of VCAM1 and ICAM1 was confirmed (Fig. 5b and Supplementary Fig. 3).

To investigate putative consequences of the EGF-regulated cytokine secretion and adhesion molecule expression, we measured further downstream parameters. Leukocyte adhesion assays showed a reduction of attached monocytes to HAoEC following EGFR activation but an upregulation of their adhesion to HCAEC (Fig. 5c). This suggests that EGF triggers not only a HCAEC-specific leukocyte accumulation but also a preventive response to it in HAoEC. Moreover, the permeability of the cell layers was measured by FITC-Dextran diffusion (Fig. 5d and Supplementary Fig. 4). The resistance of the cell layer (see “Methods” section) was slightly increased by EGFR activation in HAoEC only, suggesting a tighter layer of cells, which would then be less prone to leukocyte infiltration.

The downregulation of pro-inflammatory cytokines and adhesion molecules, alongside with the reduced leukocyte adhesion and a higher tightness of the cell layer, suggest that EGFR activation leads to a multifactorial anti-inflammatory response in HAoEC. On the other hand, the upregulation of IL-8 in HCAEC and the increased leukocyte adhesion to these cells, suggest that EGFR activation triggers a cytokine-mediated pro-inflammatory response in HCAEC.

### Unstimulated vascular EC differentially express genes associated with inflammation regulation

**Figure 6:**
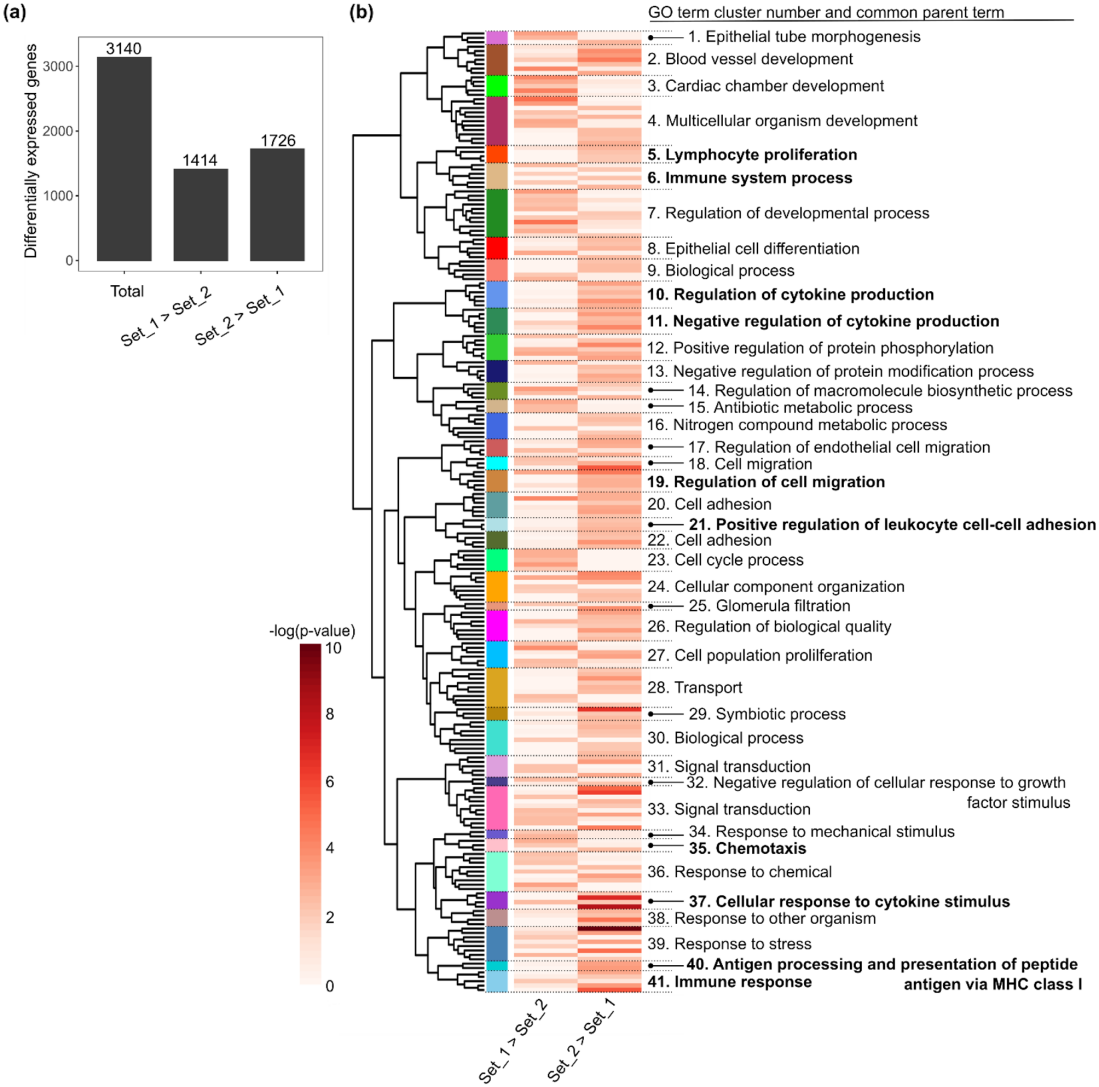
Unstimulated vascular endothelial cells differentially express genes associated with inflammation regulation. (**a**) Number of differentially expressed genes when comparing unstimulated cells from Set_1 and Set_2 (Set_1 used as a reference—number of independent replicates N = 8). (**b**) Heatmap (generated using ViSEAGO R package—see “Methods”) showing GO terms that were significantly enriched for at least one list of regulated genes used as input for the analysis. Each row corresponds to a GO term and the colour gradient to their respective − log (*p*-value) (the detailed list of GO terms and the corresponding p-values are available in Supplementary Table S4). The GO term clusters are indicated by different colours and numbers. The common parent term of the GO terms comprised in a given cluster is indicated. Those associated with inflammation regulation are highlighted in bold.

We also used our RNA-sequencing datasets to determine which cellular processes may be inherently different in distinct EC populations. To do so, we performed differential expression analysis comparing the control samples (unstimulated cells) from Set_1 (HAoEC/donor 1) to those of Set_2 (HCAEC/donor 2). 3140 genes were significantly differentially expressed. Of these 1414 genes showed a higher expression level in Set_1 than in Set_2, and 1726 a higher expression level in Set_2 than in Set_1 (Fig. 6a and Supplementary Table S1).

GO term enrichment analysis served to identify biological processes that may be favoured more in one set than in the other (Fig. 6b and Supplementary Table S4). The annotation of the GO term clusters showed that the transcriptional differences between the two sets correspond to diverse biological functions (e.g., development-related functions (clusters 1 to 4), cell cycle (clusters 22 and 27), response to stimulus (clusters 34, 38 and 39)). Several of the GO term clusters were related to inflammation- and immune reaction-regulation (e.g. production of and response to cytokine, chemotaxis and leukocyte cell–cell adhesion—highlighted in Fig. 6b). Most of the GO terms comprised in these inflammation-related clusters were exclusively enriched for genes more expressed in Set_2 than in Set_1 (Supplementary Table S4). These results therefore suggests that unstimulated cells from Set_1 and Set_2 might have different basic inflammation profiles and that those from Set_2 may have a higher inflammation-regulator potential.

### Unstimulated EC show different potential for leukocytes attraction and binding

The GO database provides information concerning the biological functions in which a given gene/protein may be involved. However, it does not indicate in which cell types the observations or predictions used for this annotation were made. We therefore sought to confirm that the genes expressed more in Set_2 than in Set_1 under basal conditions were not just genes described in immune cells but may indeed play a role in inflammation regulation in EC. To do so, we used the IPA “Diseases and Functions” tool, which has the advantage to provide the option to filter the internal database for given cell types or organs prior to the analysis (see “Methods” section). We therefore searched for enriched inflammation-related functions that may be cell type-specific, using only annotations based on experimental observations made in EC. No functions were found significantly enriched (|Z-score|≥ 2, B-H (Benjamini-Hochberg) *p*-value ≤ 0.05) with genes expressed more in Set_1 than in Set_2. On the contrary, the genes expressed more in Set_2 than in Set_1 appeared to be relevant for the adhesion of immune cells to the endothelium (Table 2), supporting the idea that the cells from Set_2 have a higher inflammatory potential.

**Table 2:** Genes more expressed in Set_2 than in Set_1 are associated with immune cell adhesion.

Diseases or functions annotation	Z-score	B-H *p*-value
Adhesion of immune cells	2.570	1.56E−03
Binding of professional phagocytic cells	2.345	2.28E−02
Binding of neutrophils	2.121	3.50E−02

Significantly enriched (|Z-score|≥ 2, B-H *p*-value ≤ 0.05) immune-related “Diseases and functions” for genes more expressed in Set_2 than in Set_1 (output of IPA). Z-scores were calculated based on annotations in the software internal database. Positive Z-scores correspond to putatively promoted functions.

The differentially expressed genes obtained when comparing Set_1 and Set_2 (Supplementary Table S1) were screened for cytokines and chemokines (using IL*, CX*, CSF*, TGFB*, IFN*, MST*, CCL* as search keys). While 4 cytokines/chemokines were expressed more in Set_1 than in Set_2, 13 pro-inflammatory cytokines/chemokines were significantly more expressed in Set_2 than in Set_1 (Supplementary Fig. 5a). Additionally, we checked for the expression of genes coding for adhesion molecules involved in endothelial integrity and leukocyte-endothelium interaction (*SELE*, *SELP*, *VCAM1*, *ICAM1* and *PECAM1). SELP* was more expressed in Set_1 than in Set_2 but *ICAM1* and *PECAM1* were more expressed in Set_2 than in Set_1 (Supplementary Fig. 5b). These results suggest that cytokine/chemokine and adhesion molecule baseline expression was different in the two sets of cells. This hints at a differential ability in leukocyte attraction and binding for the two sets of cells, and especially at a more pronounced one for cells from the Set_2.

## Discussion

With this study, we aimed to understand how the activation of the central signalling hub for vasoactive substances EGFR affects the transcriptome of endothelial cells from different types of arteries. Our RNA-sequencing analysis suggested that EGFR activation leads to an opposite regulation of inflammatory processes in aortic and coronary artery endothelial cells. The measure of relevant inflammation biomarkers in cells from multiple donors allowed us to confirm these predictions experimentally.

EGFR has been associated not only with vascular homeostasis but also with diverse pathologies, including cardiovascular diseases such as hypertension or atherosclerosis^31,32^, in which it appears mostly harmful. We previously described a role of EGFR in EC for the regulation of basal vascular function in vivo, using a transgenic mouse model. We concluded that the mouse EC-EGFR plays a minor role in vascular function and structure compared to VSMC-EGFR, but that it could still be protective during pathological situations^26^. However, we did not measure any inflammation-related parameters in that study. Our results in human cells also support the hypothesis that EC-EGFR is of vascular relevance since our both sets of EC responded to EGFR activation, including with a consequent number of regulated genes despite stringent filters applied during our RNA-sequencing analysis. We are not aware of studies reporting the effect of EGFR on the transcriptome of human EC from different vessel types nor its impact on inflammation markers in these cells. Therefore, our results suggest new functions for EC-EGFR that have not been described yet.

Nevertheless, it appeared that EGFR led to a different response in our two RNA-sequencing sets (each of them corresponding to one donor and to one EC type), including with differences in amplitude and in the nature of the regulation. Thereby, EGFR inhibited inflammation markers in the set of samples isolated from HAoEC from one single donor, while promoting them in the set of samples isolated from HCAEC from another single donor. In the first place, this suggested that the role played by EGFR in a given individual or in a given type of artery may not be generalizable. In either case, knowing that EGFR regulates EC-associated inflammation is highly relevant when considering the role of the latter in cardiovascular diseases, such as atherosclerosis or coronary artery diseases. Studies have indeed shown that inflammation plays a key role in the development of such pathologies^33^. For instance, the first stages of atherosclerosis consist in the development of fatty streaks, an accumulation of lipid-laden cells beneath the endothelium. The recruitment and the infiltration of immune cells is thus a critical step in the development of this pathology.

The measurement of selected inflammation markers (by immunoassays or Western Blots) on cells from multiple donors nonetheless allowed us to clarify the cause behind the different responses to EGFR activation observed at the transcriptomic level. Indeed, each of these markers was regulated in the same direction in all samples of a given cell type, no matter the donor origin. This indicates that the differential response to EGFR activation with regards to inflammation-related processes was mostly attributable to the differences between the vessels. Of course, in future studies, more studies have to be included to substantiate this hypothesis.

We report that EGFR activation leads to an increased IL-8 secretion by HCAEC. This is consistent with the observed increase in monocyte-HCAEC interaction, since the chemokine IL-8 primarily recruits monocytes and is critical for the monocyte-endothelium interaction^30^. On the other hand, IL-8 also has angiogenetic properties, which may contribute to the formation of atherosclerotic plaques^34^. The activation of HCAEC-EGFR thus upregulates a major key player in coronary artery diseases, which supports the idea that it triggers a pro-inflammatory endothelial phenotype in coronary arteries.

Interestingly, HAoEC-EGFR activation leads to opposite effects, including the downregulation of MCP-1 secretion and adhesion molecules expression, whose strong expression is commonly associated with cardiovascular diseases such as atherosclerosis^30,35^. Moreover, these results suggested an EGF-induced decrease of leukocyte recruitment by HAoEC since MCP-1, VCAM-1 and ICAM-1 are key players in this process^29,30^, and we could show that EGF actually induced a reduction of monocyte-HAoEC interaction. The HAoEC layer also tightened after EGF treatment, suggesting that HAoEC are not only less prone to bind to immune cells but also to let them go through their barrier. The HAoEC-EGFR may thus play a protective anti-inflammatory role in the aorta.

Our bioinformatics comparative analysis based on multiple biological replicates thus allowed the establishment of a statistically sound hypothesis concerning the divergence of aortic and coronary EC with regards to their responsiveness to EGFR activation. By experimentally confirming the effect of EGFR activation on cells from multiple donors, we confirmed the predicted dual role of this receptor is donor-independent in female endothelial cells. As previously mentioned, it has been described that conductive and resistant arteries from which HAoEC and HCAEC are isolated, respectively, display different responses and susceptibilities to pathological situations^18^. Our evidences that different inflammation profiles can be induced by EGF in the two cell types is therefore an important step to understand the differential pathology development in the corresponding vessels. However, as previously mentioned, endothelial cells show inherent transcriptional sex-differences, including for genes related to immune-response^13,15^. This means that our conclusions would need to be tested in male vascular endothelial cells before being extended.

Furthermore, the EGFR signaling pathway is not strictly linear as its activation can be achieved not only by EGF-binding but also by transactivation by receptors for other vasoactive substances^20^. We already showed that the impact of EGFR activation on gene expression is modulated by Angiotensin II and Thromboxan A2 in HEK cells and murine aortic VSMC, respectively^27,36^. This aspect shall be considered in a second phase of our study, to know how a more physiological-like situation actually affects primary vascular EC.

We also attempted to determine the inherent differences between unstimulated arterial EC and the results suggest variable inflammatory potentials. Only few studies have investigated heterogeneity of human EC from large vessels. For instance, Ho et al.^10^ performed a microarray analysis to determine if different types of quiescent EC, including HAoEC and HCAEC, had different transcriptomic profiles. They could distinguish HAoEC and HCAEC (single male donors) by a certain number of genes, including *CCL2* (MCP-1), which was more expressed in HCAEC than HAoEC. However, the microarray approach, mostly targeted on “endothelial-enriched” genes here, introduced a bias that prevented further functional analysis and the putative identification of more inflammation-related genes. More recently, Nakato et al.^8^ performed RNA-sequencing and ChIP-Seq on 9 vascular cell types, including HAoEC and HCAEC (mixed multiple donors). They could show that EC have different transcriptomic and epigenetic profiles, mostly depending on where the cells were isolated from. They also identified some inflammation-related genes by clustering genes differentially expressed throughout the different EC. Finally, Hu et al. applied single cell RNA-sequencing on non-diseased (without calcification or stenosis) human cardiac arteries, including aorta, coronary and pulmonary arteries, from three different patients (unmentioned sex)^12^. They showed that the largest subpopulation of EC in each of these different vessels expressed inflammatory genes and were involved in the regulation of inflammation. It is, however, not clear if the pro-inflammatory EC expressed these inflammation makers at the same level in all artery types.

Although our RNA-sequencing data analysis is based on data from a limited number of donors (one female donor per cell type), it also suggests that EC from the aorta and coronary arteries may have inherently different inflammation profiles. At this point, we cannot distinguish if the observed differences are due to inherent differences between the two donors or to actual differences between HAoEC and HCAEC. However, based on previous published studies and on our results showing that EGF induces opposite inflammation regulation in the two cell types, we hypothesize that female HAoEC and HCAEC have different inflammation profiles and potentials under basal conditions already. But this still needs to be experimentally tested using cells from multiple female donors in order to confirm that our predictions were not affected by different donor-associated EC inflammatory states. Indeed, it has been reported that the latter are influenced by multiple factors, including infections and local immune reactions, but also pathologies such as hypertension, diabetes or obesity^18,37^. This will be considered in further studies since gaining a better overview of inherent differences between unstimulated endothelial cells would be highly beneficial to better understand the variable susceptibility of vessel regions to vascular diseases^38,39^, especially in female individuals.

To summarize, this present study confirms that vascular endothelial cells are far from being uniform, as female HAoEC and HCAEC show strong differences concerning their responsiveness to EGFR activation. EC-EGFR seems to be a relevant but Janus-faced modulator of pathological processes, which is protective in some but not all vessel types during inflammatory processes. Thereby, EC-EGFR can modulate vascular dysfunction, vascular remodelling or the development of atherosclerosis. Although already abundantly described, and this often as being harmful, EGFR appears to still have secrets that need to be unravelled.

## Methods

### Buffers, chemicals and antibodies

Buffer compositions and references/providers of chemical, kits, cells, cell culture media and antibodies are listed in Supplementary Methods.

### Cell culture

Commercially available HAoEC (cells isolated from the thoracic aorta of 2 adult female donors—38 and 61 y.o.—no pathology documented) and HCAEC (cells isolated from 2 adult female donors—55 and 51 y.o.—no pathology documented) were acquired (see Supplementary Methods for references and provider details) and cultivated with “Endothelial Cell Growth Medium MV2” supplemented with “Endothelial Cell Growth Medium MV2 SupplementMix” (Final concentrations after addition to the medium: 5% FCS, 5 ng/mL EGF, 10 ng/mL bFGF, 20 ng/mL IGF-1, 0.5 ng/mL VEGF, 1 µg/mL ascorbic acid and 0.2 µg/mL hydrocortisone). Before all experiments, cell synchronization and quiescence (to reach a physiological-like situation) were induced by 24 h in media with minimal supplementation (“Endothelial Cell Growth Medium MV2” with 1% HSA, 1 µg/mL ascorbic acid and 0.2 µg/mL hydrocortisone only). This media was also used for further incubation with 10 ng/mL EGF. All cells used for the diverse experiments underwent less than 8 cell culture passages and were > 90% confluent before the start of the incubation.

### RNA and protein sample preparation

Total RNA and proteins were isolated after 48 h treatment with BlueZol Reagent as described in the user manual. The RNA samples were treated with “Turbo DNAse-free kit” (following the “rigorous DNAse treatment” protocol from the manufacturer) to remove eventual genomic DNA contaminations and were cleaned by ethanol precipitation (with 3 M sodium acetate, glycogen and 100% ethanol). The RNA concentration was determined by NanoDrop (Biochrom, Germany). The quality of the to-be-sequenced RNA samples was assessed using a 2100 Bioanalyzer (Agilent Technologies, Germany) and all samples had a RNA Integrity Number (RIN) above 7 (with 10 as maximal possible value). The protein samples were resuspended in 1% SDS and the concentrations were determined by BCA assay.

### RNA sequencing

Two sets of RNA samples (from one donor for each cell type) were sequenced (see Supplementary Methods for donor information). Novogene Co., Ltd (Cambridge, United-Kingdom) carried out the sequencing libraries preparation (poly(A) enrichment) and the paired-end sequencing (2 × 150 bp) runs on a NovaSeq6000 Illumina system (N = 8). Adaptor clipping and data quality control was provided by the service company as well.

Read mapping to the human genome *hg38* was done with HISAT2^40^ (v. 2.1.0) and featureCounts^41^ (2.0.0, –M –t exon) was used to count the mapped reads. Gene annotation was done using BiomaRt^42^ (v.2.44.4) to access Ensembl archive v101. Raw RNA sequencing data and annotated counts are publicly available on Gene Expression Omnibus (GEO) database (https://www.ncbi.nlm.nih.gov/geo). GEO accession number: GSE206410.

### Differential expression analysis

Differential expression analysis was performed using edgeR^43^ (3.30.3) and DESeq2^44^ (1.28.1). The multi-variable design ~ cellPassage + set_treatment was used for both tools to overcome the variation induced by the different cell culture passages (Supplementary Fig. 1). Genes with sufficient counts to be considered in the statistical analyses were filtered using the filterByExpr edgeR function and the independent filtering parameter (α = 0.05) of the DESeq2 results function. Normalization factors were calculated with the “trimmed mean of M value” (TMM) method in the edgeR analysis. Significantly “differentially expressed genes” were defined as genes with a false discovery rate (FDR) below 0.01 in both DESeq2 and edgeR outputs (overlap of the respective results), with at least 5 FPM on average in one of the sample groups considered for a given comparison and with |log2 Fold Change|≥ 0.32 (threshold based on the inherent variation in control samples, corresponds to a 25% change—Supplementary Table S4).

### Gene ontology enrichment analysis

The source code of the open source ViSEAGO^45^ R package (1.2.0) was adapted to perform Gene Ontology (GO) term enrichment analysis and data visualization. Shortly, it used topGO^46^ (2.40.0) to perform GO analysis (GO annotation accessed with Ensembl v101—parameters: algorithm = “weight01”, statistic = “fisher”, ont = “BP”). GO terms were defined as significantly enriched if *p*-value ≤ 0.01 and enrichment *E* ≥ 3, with *E* = (intersection size/query size)/(term size/effective domain size). GO terms clustering was done with the dynamicTreeCut R package (version 1.63-1, https://cran.r-project.org/package=dynamicTreeCut), using “Semantic Similarity Distances” as distance matrix (calculated with “Wang” method) between the GO terms and the corresponding hierarchical clustering dendrogram (output of hclust, “ward.D2” method). The common parent term of GO terms included in a given cluster was identify by common ancestor mapping.

### Ingenuity pathways analysis

To comprehend the impact of differential gene expression on inflammation regulation by EC, we used the “Diseases and Functions” (identify downstream effects) and “Regulator effects” (links downstream effects analysis and putative upstream regulators) tools from QIAGEN Ingenuity Pathway Analysis^47^ (IPA https://digitalinsights.qiagen.com/IPA). The following analysis settings were used: (1) the Ensembl identifiers of the regulated genes were mapped to networks incorporated into the software database; (2) only IPA annotations based on previous experimental observations in human or mouse were included; (3) for the analysis concerning the genes more expressed in Set_2 than in Set_1 (untreated samples only), only annotations based on findings in endothelial cells were included. Enriched “Diseases and Functions” were filtered for functions linked to inflammation regulation (keys for categories filtering: include “*immu*, *infla*”; exclude “*cancer*, *disease*”) and the results for analyses on EGF-regulated genes were matched using the featured “Comparison Analysis” option.

“Regulator effects” networks were generated for “Diseases and Functions” related to “Immune cell trafficking” (*p*-value ≤ 0.001—based on the results of the comparison analysis) with putative upstream regulators (include cytokine, enzyme, GPCR, growth factor, ion channel, kinase, ligand-dependent nuclear receptor, peptidase, phosphatase, transcription regulator, translation regulator, transmembrane receptor and transporter) displaying a |Z-score| ≥ 2 and a *p*-value ≤ 0.001. Only networks with a consistency score CS > 0 were considered. Network edges directed from upstream regulators to regulated genes were filtered for those corresponding to “expression regulation”.

### Western blot

For each sample, 40 µg of proteins were denaturated with 6 × Laemmli Buffer for 30 min at 37 °C. Proteins were separated by 10% SDS-PAGE and transferred onto 45 µm nitrocellulose membranes. Free binding sites of the membrane were blocked with a 5% solution of non-fat dry milk in TBS-Tween. The membranes were incubated overnight with primary antibodies diluted in 5% BSA in TBS-Tween. EGFR, VCAM1, ICAM1, PECAM1, GAPDH and HSP90 were detected using IRDye-couple fluorescent secondary antibodies (diluted in 5% solution of non-fat dry milk in TBS-Tween) and an Odyssey imaging system (LI-COR Biosciences, Germany). Densitometry analysis was performed with Quantity One software (version 4.6.9, BioRad, Germany). GAPDH and β-actin were used as reference for relative quantification.

### ELISA for 5-bromo-2’-deoxyuidine

ELISA for BrdU was performed as previously described^36^. Shorty, EC were cultivated close to confluency in a 96-well plate and incubated with 10 µM (final concentration) BrdU and 10 µg/L EGF. Cells incubated with BrdU and supplemented media (see "Methods" 2.2) served as positive control. After 48 h incubation, BrdU-incorporation was quantified using mouse anti-BrdU antibody and anti-mouse IgG HRP-linked antibody. The cell density of each well was estimated by 0.2% Trypan Blue staining. The values for each independent replicate correspond to the mean of 6 wells measured per treatment condition.

### Permeability assay

Confluent HAoEC or HCAEC were cultivated onto cell-culture inserts in 6 well-plates (0.4 µm—Sarstedt, Germany). Cell-culture inserts without cells served as positive control. After 24 h incubation with or without EGF, fluorescein isothiocyanate (FITC)-Dextran 70 kDa was added to the lowest compartment (final concentration: 1 g/L). The cells were placed back at 37 °C, 5% CO_2_. 20 µL of the cell culture media were collected from the top of the cell culture insert after 3 h, 6 h and 24 h. After dilution with 100 µL HEPES-Ringer buffer, FITC fluorescence (excitation/emission: 480 nm/520 nm) was measured with an Infinite M200 plate-reader (Tecan, Germany). The resistance to FITC-Dextran diffusion (generated by the cell layers) was calculated as follows: (1/diffusion rate for cell culture insert with cells)—(1/diffusion rate for cell culture insert without cells) for each replicate and each treatment, with the diffusion rate (fluorescence/time) of FITC-Dextran between 0 and 6 h (linear phase).

### Immunoassay for IL-8 and MCP-1

The kits “ProQuantum Human IL-8 Immunoassay Kit” and “ProQuantum Human MCP-1 Immunoassay Kit” (Invitrogen, Germany) served to measure the concentration of secreted CXCL8/IL-8 and CCL2/MCP-1, respectively. The measurements were performed following the manufacturer’s instructions on cell culture media harvested prior to RNA and protein isolation (see "Methods" 2.3). The amounts of secreted cytokine were normalized to the total amount of protein in the corresponding protein sample.

### Leukocyte adhesion assay

EC were cultivated close to confluency in a 96-well plate and incubated with 10 µg/L EGF. After 48 h incubation, the nuclei were stained with 0.5 µg/mL Hoechst 33342 in HEPES-Ringer buffer, at 37 °C. Meanwhile, THP-1 cells (human monocytic cell line, cultivated with RPMI media with 10% FCS) were stained with 2 µM calcein-AM in HEPES-Ringer buffer, for 30 min at 37 °C. THP-1 cells were washed by successive centrifugations with 1xPBS and then counted with a CASY cell counter system (Innovatis, Germany). The Hoechst 33342-dye solution was removed from the EC and 0.5 × 10^6^ THP-1 cells/mL (diluted in HEPES-Ringer buffer) were added onto the EC. The plate was incubated for 60 min at 37 °C and was then washed with 1xPBS. The Hoechst 33342 (excitation/emission: 358/461 nm) and calcein (352/461 nm) signals were detected by digital fluorescence microscopy (Cytation3—BioTek, Germany) and served to quantify the number of EC and monocytes, respectively. Data analysis was performed with the Gen5 2.09 software (BioTek, Germany).

### Statistical analysis for experimental validation data

For all experiments other than RNA-sequencing, significant differences in between groups was assessed by Wilcoxon (rank sum) test (*p* < 0.05) and *Chi-*squared test for outlier removal was performed with outliers R package (https://cran.r-project.org/package=outliers).

### Supplementary Information

Supplementary Figures.

Supplementary Information.

Supplementary Table S1.

Supplementary Table S2.

Supplementary Table S3.

Supplementary Table S4.

Supplementary Table S5.

## Data Availability

RNA sequencing data on which is based this study are available online in the GEO database with the accession number GSE206410. All other datasets used during the current study are available from the corresponding author on reasonable request.
