# Supplementary material for: EGFR activation differentially affects the inflammatory profiles of female human aortic and coronary artery endothelial cells
Source: Sci Rep. 2023 Dec 20;13:22827. doi: 10.1038/s41598-023-50148-7 (PMC10739936; doi:10.1038/s41598-023-50148-7)
Supplement: Supplementary file 1 — Supplementary Figures. [file 41598_2023_50148_MOESM1_ESM.pdf]

SUPPLEMENTARY FIGURES

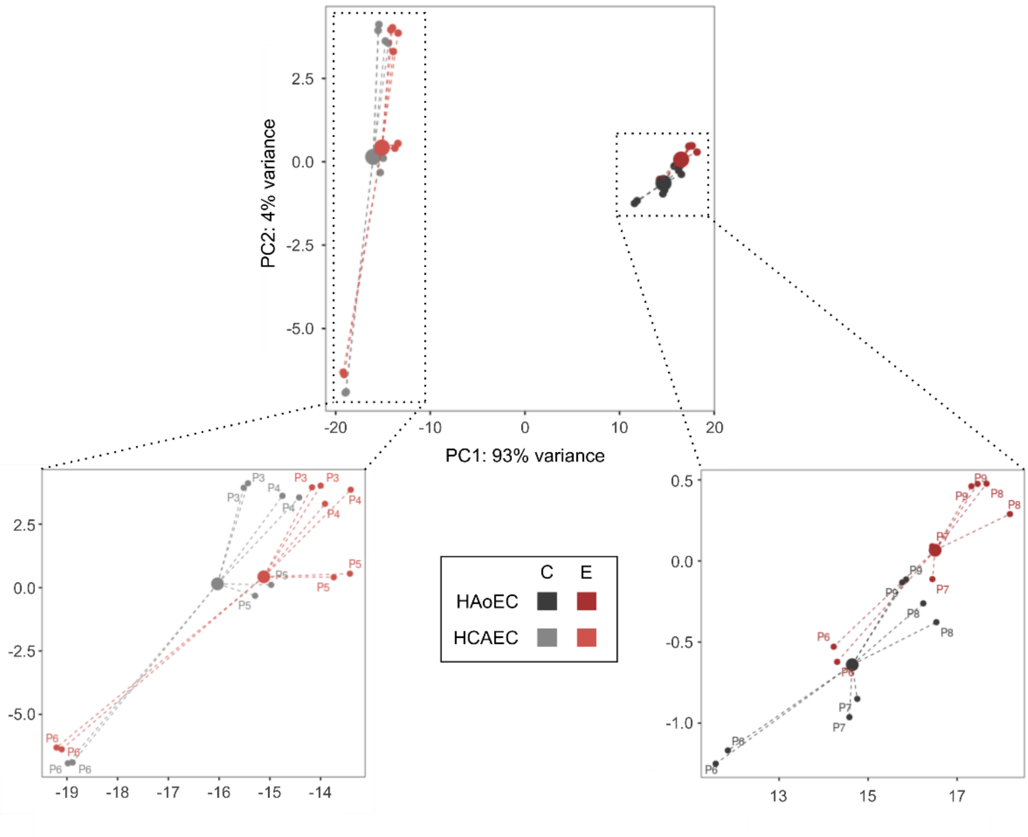

Supplementary Figure 1: Principal component analysis reveal a combination of cell type-, treatment- and cell culture passage-effects. PCA was performed on all samples considered for the analysis. Principal components 1 and 2 (PC1 and PC2) are displayed here. PC1 appears to correspond to the cell type. A close-up on the samples from each of cell-type are shown. Here variances induced both by EGF-treatment and cell culture passage (indicated by Px, with x the passage number) are visible. The multi-variable design ~ `cellPassage + celltype_treatment` was therefore used for differential expression analyses.

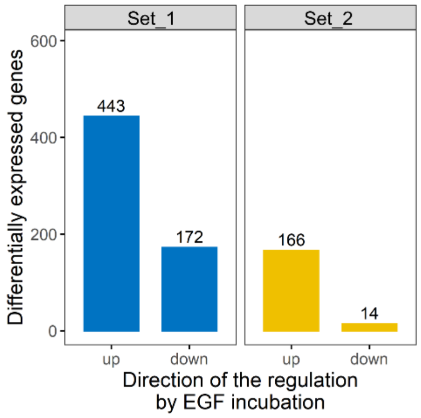

Supplementary Figure 2: Regulation direction of the genes regulated by EGF. Number of up- (log2 fold change > 0) and down- (log2 fold change <0) regulated genes following EGF incubation, in Set_1 and in Set_2.

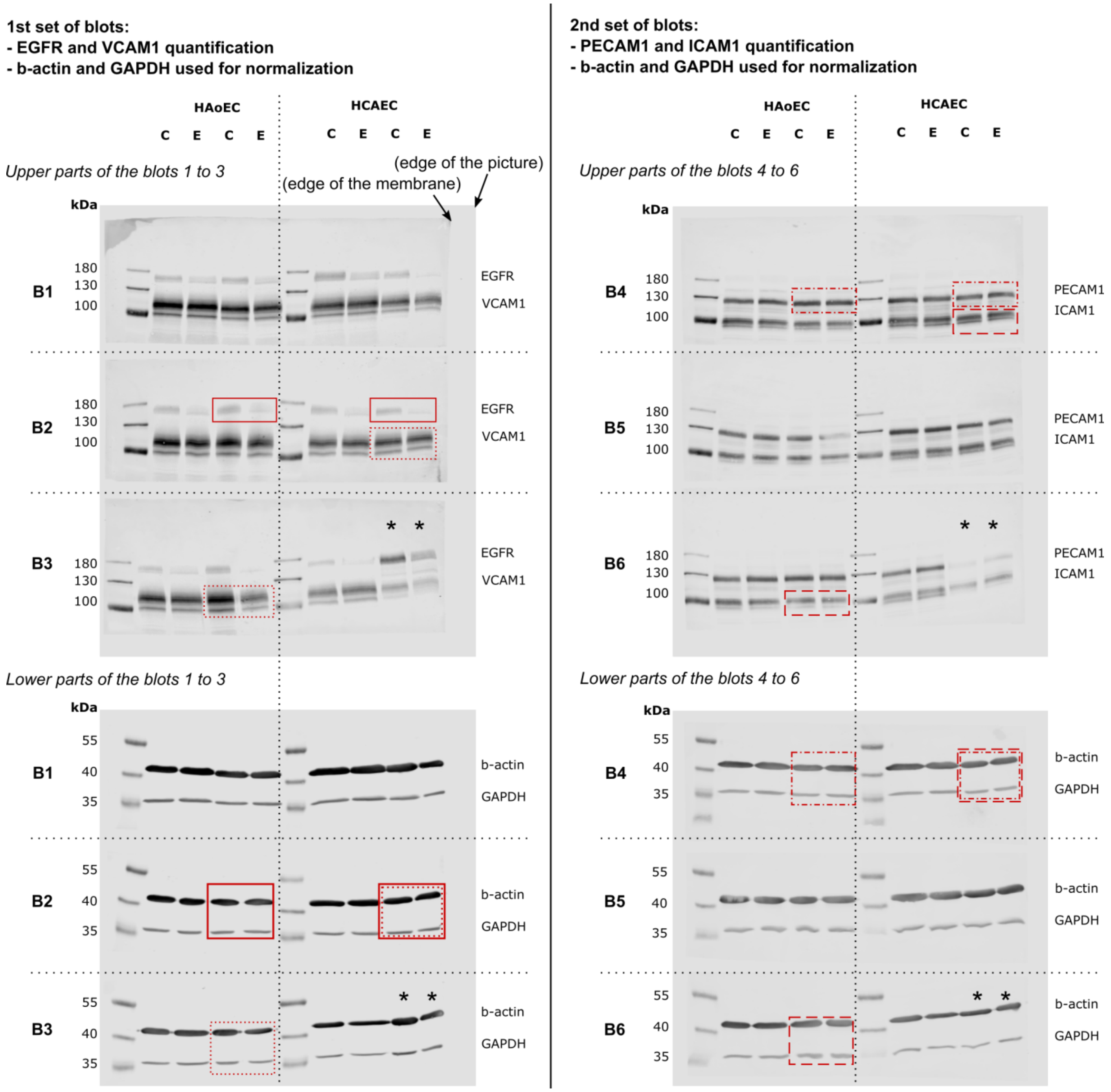

Supplementary Figure 3: Western Blot membranes. Original pictures considered for the quantification of VCAM1 and EGFR (left); PECAM1 and ICAM1 (right).The red boxes indicated which bands are shown in the Figures 1 and 5 (each line type corresponds to one target). Two sets of experiments were performed on the same samples: (1) for EGFR and VCAM1 quantification (Blots (B) 1 to 3) and (2) for PECAM1 and ICAM1 quantification (B4 to B6). For each experiment, 3 blots were prepared, each of them including 2 independent replicates (with control and EGF-incubated samples) per cell type (Final number of replicates for each target and per cell type N = 6). Membranes were cut prior to the incubation with primary antibodies (upper parts were incubated with antibodies against EGFR/VCAM1 or PECAM1/ICAM1, lower parts with antibodies against β-actin/GAPDH). For each experiment, all membrane pieces that were incubated with the same set of antibodies were detected at the same time (what means that, for each blot batch, a single picture (.tif file) was generated by our Odyssey imaging system (LI-COR Biosciences) that is shown here).

Samples marked with an asterisk (*) were not considered for the final analysis as they were identified as outlier by Chi-squared test.

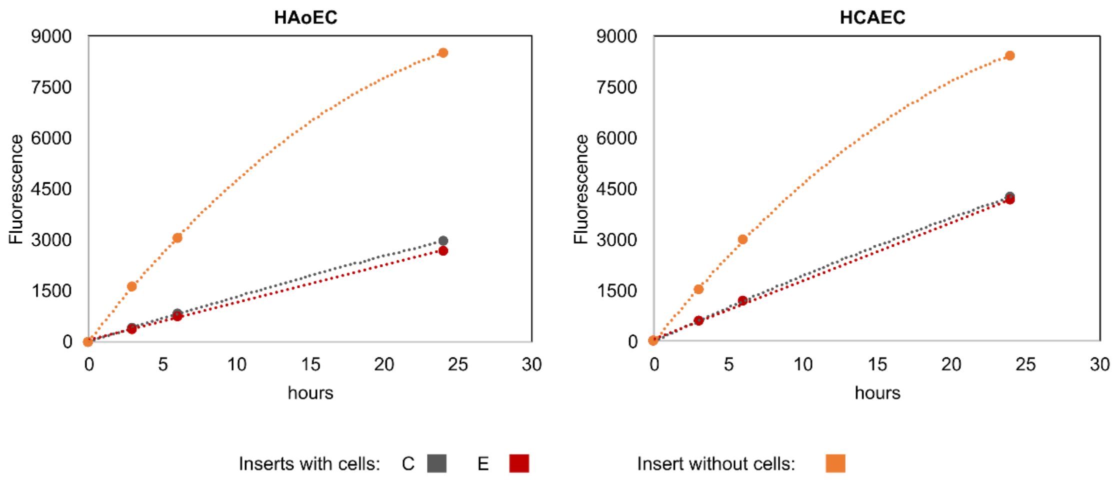

Supplementary Figure 4: HAoEC and HCAEC monolayers slow down the diffusion of FITC-Dextran. Insert without cells were used as positive controls for the permeability assay, to check if the cultivation of EC onto the cell culture inserts indeed led to a reduction of FITC-Dextran diffusion. The fluorescence of the cell culture media on contained within the cell culture insert was measured after 3h, 6h and 24h.

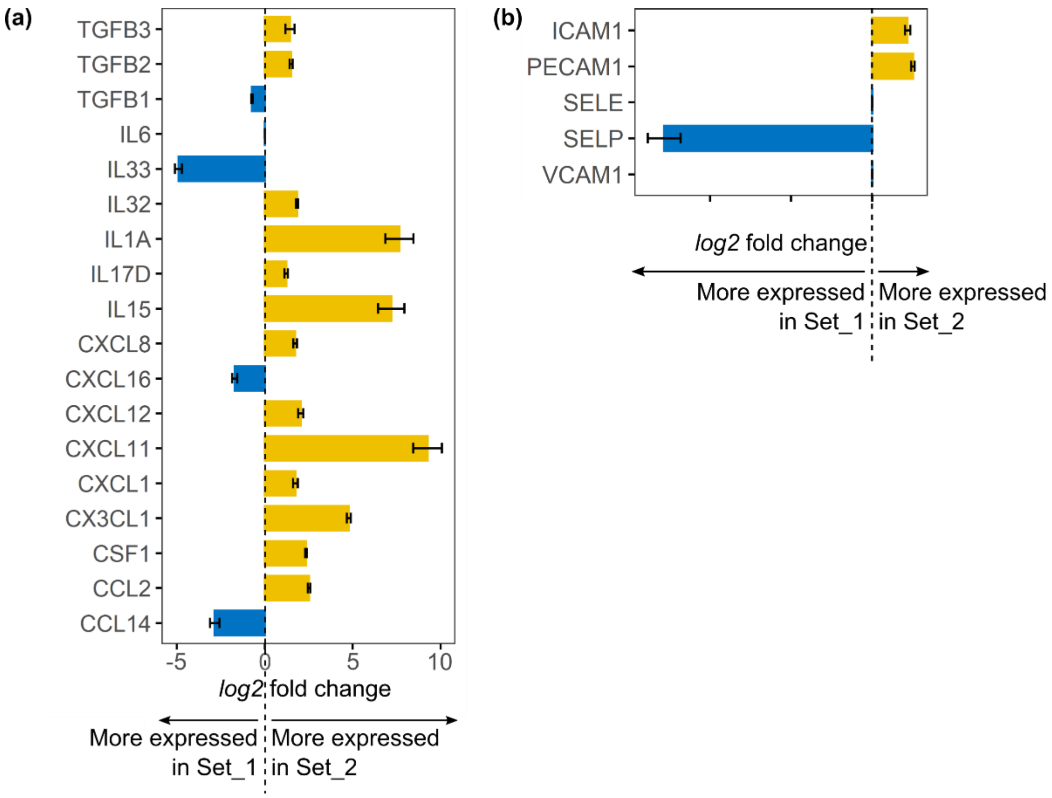

Supplementary Figure 5: EC have different basal expression level of cytokines/chemokines and adhesion molecules. Log2 fold changes (calculated by DESeq2, error bars show the standard errors) for significantly differentially expressed (a) cytokines/chemokines and (b) selected adhesion molecules (right) when comparing the unstimulated samples from Set_1 to those of Set_2 (Set_1 used as reference) (Supplementary Table 2).
