# Supplementary material for: EGFR activation differentially affects the inflammatory profiles of female human aortic and coronary artery endothelial cells
Source: Sci Rep. 2023 Dec 20;13:22827. doi: 10.1038/s41598-023-50148-7 (PMC10739936; doi:10.1038/s41598-023-50148-7)
Supplement: Supplementary file 2 — Supplementary Information. [file 41598_2023_50148_MOESM2_ESM.pdf]

SUPPLEMENTARY METHODS

Cell culture

Buffer name	Composition
Endothelial Cell Growth Medium MV2	PromoCell, Germany (#C-22221)
Endothelial Cell Growth Medium MV2 SupplementMix	PromoCell, Germany (#C39226)
Human aortic endothelial cells	PromoCell, Germany (#C-12271, 2 donors with the lot numbers: 431Z013.5 (used for RNA-sequencing and experimental validation) and 458Z035.1 (experimental validation))
Human coronary artery endothelial cells	LONZA, USA (#CC-2585, lot number: 18TL165227 – used for RNA-sequencing and experimental validation) and PromoCell, Germany (#C-12221, lot number: 458Z016.11 – used for experimental validation)
RPMI	PAN-Biotech, Germany

Chemical and kit providers

Chemical	Provider
Ascorbic acid	Merck, Germany
BlueZol	Serva, Germany
BrdU	Sigma, Germany
Calcein-AM	Biomol, Germany
EGF	Merck, Germany
FCS	Pan Biotech, Germany
FITC-Dextran	Sigma, Germany (#SLCB8500)
Hoechst 33342	ThermoFisher Scientific, Germany
HSA	Sigma, Germany
Hydrocortisone	Sigma, Germany
SuperScript II reverse transcriptase	Invitrogen, Life Technologies, Germany
Trypan Blue	Life Technologies, Germany

Kit	Provider
Turbo DNAse-free kit	Invitrogen, Life Technologies, Germany
**Kit**	**Provider**
ProQuantum Human IL-8 Immunoassay Kit	Invitrogen, Germany
ProQuantum Human MCP-1 Immunoassay Kit	Invitrogen, Germany

Buffer compositions

Buffer name	Composition
Blocking Solution	0.1% Triton X-100 in PBS, 10% FCS
HEPES-Ringer buffer	122.5 mM NaCl, 5.4 mM KCl, 0.8 mM MgCl2·6H2O, 1.2 mM CaCl2·2H2O, 1 mM NaH2PO4·H2O, 5.5 D-glucose, 10 mM HEPES, pH 7.4
HRP-substrate	11.8 mg/mL Sodium phosphate dibasic dehydrate, 7.3 mg/mL Citric Acid, 0.5 mg/mL o-Phenylenediamine, 0.015% H2O2
Laemmli Buffer	0.5 M Tris pH 6.8, 10% SDS, 10% Gylcerol
Permabilization Buffer	0.1% Triton X-100 in PBS, 37 mg/L Na-Orthovanadate
TBS	17 mM Tris-HCl, 3 mM Tris-base, 140 mM NaCl, pH 7.4 HCl
TBS-Tween	TBS with 0.1% Tween-20

Antibodies

Target antigen	Source	Catalog #	Working conc.
EGFR	Cell Signaling Technology	4267	1:500
ICAM1	Cell Signaling Technology	67836	1:500
VCAM1	Cell Signaling Technology	13662	1:500
PECAM1	Santa Cruz Biotechnology	Sc-1506-R	1:500
GAPDH	Cell Signaling Technology	97166	1:2 000
b-actin	Cell Signaling Technology	3700	1:2 000
Anti-Mouse IRDye 680RD	LICOR Biosciences	926-68070	1:20 000
Anti-Rabbit IRDye 800CW	LICOR Biosciences	926-32211	1:20 000
BrdU	Becton Dickinson	347580	1:200
Anti-mouse IgG, HRP-linked	Cell Signaling Technology	7076	1:1 000

Abbreviations within Methods

BSA Bovine serum albumin

ELISA	Enzyme-linked immunosorbent assay
FCS	Fetal calf serum
HEPES	4-(2-hydroxyethyl)-1-piperazineethanesulfonic acid
HRP	Horseradish peroxidase
HSA	Human serum albumin
IgG	Immunoglobulin G
PBS	Phosphate-buffered saline
RIN	RNA integrity number
SDS-PAGE	Sodium dodecyl sulfate-polyacrylamide gel electrophoresis
TBS	Tris-buffered saline
TMM	Trimmed mean of M value
